# Non-dipping blood pressure pattern is associated with cardiovascular events in a 21-year follow-up study

**DOI:** 10.1038/s41371-024-00909-2

**Published:** 2024-04-03

**Authors:** Päivi A. Lempiäinen, Antti Ylitalo, Heikki Huikuri, Y. Antero Kesäniemi, Olavi H. Ukkola

**Affiliations:** 1 Medical Research Center Oulu, Oulu University Hospital and Research Unit of Biomedicine and Internal Medicine, Faculty of Medicine, University of Oulu, Oulu, Finland; 2 Heart Center, Turku University Hospital and University of Turku, Turku, Finland

**Keywords:** Risk factors, Cardiovascular diseases

## Abstract

Non-dipping blood pressure (BP) pattern is a predictor for cardiovascular (CV) events and mortality. We evaluated dipping status change and its association with incidence of non-fatal CV events in middle-aged subjects. The OPERA study was carried out during the years 1991–1993, with a follow-up study 21.7 years later. In this study, we included 452 participants with 24-h ambulatory BP measurements (ABPM) available in both surveys. The study population was divided into four groups according to the dipping pattern change: dipping–dipping (*n* = 152/33.6%), dipping–non-dipping (*n* = 198/43.8%), non-dipping–dipping (*n* = 20/4.4%), and non-dipping–non-dipping (*n* = 82/18.1%). Sixty-five participants experienced a CV event (14.4%) during the 21.7 (SD 0.8) years of follow-up. The incidence of events was highest (28%) in the non-dipping–non-dipping group, and lowest (6.6%) in the dipping–dipping group (*p* < 0.001). In Cox regression analyses the covariates were age, sex, total cholesterol, hypertension and use of antihypertensive medication, systolic office BP and ambulatory mean or nighttime systolic BP, as well as the change in the variables during the follow-up period. After adjustments, the association of the non-dipping–non-dipping pattern with CV events compared with the dipping–dipping pattern remained significant (HR 4.01; 95% CI 1.89–8.67, *p* < 0.001). In summary, non-dipping–non-dipping pattern was associated with non-fatal CV events in the long term, and the effect was independent of the conventional risk factors including office and ambulatory BP levels.

## Introduction

Ambulatory blood pressure measurement (ABPM) is a method which usually provides 24-h assessment of blood pressure (BP) readings and its diurnal variation in a domestic setting during normal daily activities. Recent guidelines recommend ABPM for screening of masked hypertension, detecting white coat hypertension, for diagnosing hypertension, and as the most accurate method for assessing cardiovascular (CV) risk and for detecting nocturnal hypertension [1, 2]. BP dipping is a physiological phenomenon in which nighttime BP declines 10–20% compared with daytime BP levels. In a proportion of individuals, however, the normal circadian BP variation is inadequate, and the decrease in BP is therefore less than 10%. This is considered a non-dipping BP pattern, which was first described by O’Brien et al. [3]. The non-dipping phenotype has been shown to be an independent risk factor for CV events in hypertensive patients and in different populations [3–7]. Non-dipping has also been associated with metabolic abnormalities [8], end organ damage [7, 9, 10] and new-onset diabetes [11]. Furthermore, some studies have suggested that nocturnal BP levels, sometimes accompanied by a non-dipping pattern, are better at predicting CV events, CV mortality and total mortality than office BP [12].

Earlier studies on ambulatory BP characteristics have been mostly based on the risk of outcomes associated with only one monitoring. However, only limited information is available regarding the long-term consistency of the non-dipping pattern. In this study we present long-term data on the changes of ABPM characteristics and the association of these changes with CV morbidity. To the best of our knowledge, there are no prior studies with follow-up data that are as long and with baseline examinations that are as detailed as in the present study.

## Methods

### Study population

This research is part of the OPERA (Oulu Project Elucidating Risk of Atherosclerosis) project, a population-based cohort study designed to evaluate the risk factors and endpoints of atherosclerotic CV disease. The Ethics Committee of the Faculty of Medicine, University of Oulu approved the OPERA study, and it was conducted by the principles of the Declaration of Helsinki. All study subjects gave an informed consent. The details of the study population and the selection criteria have been published earlier [13]. In short, 600 hypertensive men and women aged 40–62 years, were randomly selected from the register of reimbursement for hypertension medication. In addition, an age- and sex-matched cohort of 600 normotensive subjects was randomly sampled from the social register of the area.

### Baseline study

Out of the 1200 individuals invited, 1045 (87.1%) participated in the baseline study during the years 1990–1993. The participants attended a clinical examination and answered a questionnaire, and laboratory samples were drawn after fasting [13]. The office BP was measured by a registered study nurse, using an appropriately fitted cuff size and an automatic oscillometric device (Dinamap Procare 100, Criticon, Tampa, Florida, USA) [14]. BP was measured three times in a sitting position, after a rest of a minimum of 5 min, with 1-min intervals between the measurements. The mean of the second and the third measurement was used for the analyses.

ABPM was recorded in 903 participants by a noninvasive fully automatic SpaceLabs 90207 oscillometric unit (SpaceLabs Inc., Redmond, Washington, USA). The measurements were taken every 15 min from 04:00 a.m. to midnight, and every 20 min between midnight and 04:00 a.m. The British Hypertension Society and the US Association for the Advancement of Medical Instrumentation have previously confirmed the accuracy and reproducibility of the BP readings acquired with this device [15]. In each individual, the proper positioning of the cuff was assessed by means of the similarity (difference < 5 mmHg) between four SpaceLabs BP measurements and four auscultatory readings using a Y-connector. Values were automatically excluded from the analysis if systolic BP (SBP) was <70 or >250 mmHg, or if diastolic BP (DBP) was <40 or >150 mmHg, or if the heart rate was <40 or >150 beats per minute. Less than 3% of the BP readings were rejected as artifacts based on these criteria [16]. Further details of the ABPM of the baseline study can be found in a previous publication [17].

### Follow-up study

The follow-up study was carried out during the years 2013 and 2014, and 600 subjects took part in it. ABPM was completed in 483 of the original participants (53.5%) of which 30 were excluded for a previous CV event and 1 for missing values, leaving 452 recordings for analyses. During the follow-up period, the ABPM setting was similar compared with the baseline measurement, and the recordings were made with Oscar 2 oscillometric ABP monitor (SunTech Medical) [18]. The mean follow-up time between the recordings was 21.7 ± 0.8 years.

### Follow-up of cardiovascular events

Follow-up time of the CV events was defined as the time from the date of the baseline examination to the first non-fatal CV event or until December 31, 2014. The endpoint was the first CV event: a major coronary heart disease (CHD) event or stroke, whichever occurred first. The data on CV events was obtained by the ICD (the International Classification of Diseases) codes from national registries, the Hospital Discharge Register and the Care Register for Health Care of the Finnish Institute for Health and Welfare (THL). Each study subject was identified by a personal identity code. The diagnoses were classified according to the ICD Ninth Revision (ICD-9) before 1996 and the Tenth Revision (ICD-10) thereafter. The ICD-10 code was mandated for use in Finland from January 1, 1996. CHD was based on the following diagnoses: I20, I21, I22 (ICD-10)/410, 4110 (ICD-9) as the main diagnosis, and I21, I22 (ICD-10)/410 (ICD-9) as a first or second side diagnosis or third diagnosis (ICD-9 only), or coronary artery bypass grafting, or coronary angioplasty. Stroke as an endpoint included I61, I63 (not I63.6), I64 (ICD-10) and 431, 4330A, 4331A, 4339A, 4340A, 4341A, 4349A, 436 (ICD-9) as the main diagnosis or as a side diagnosis.

### Statistical methods

Data analyses were performed with IBM SPSS Statistics for Windows version 27.0 (IBM Corp. IBM SPSS Statistics for Windows. Armonk, NY: IBM Corp; 2020). A *p* value < 0.05 was considered as statistically significant. Baseline data are expressed as prevalence, mean ± SD for continuous variables, or median with 25th and 75th quartiles for skewed variables. Continuous variables were tested for difference between the CV event or non-event groups with Student’s *t* test or with Mann–Whitney’s test, when appropriate. Log transformation was performed for skewed variables for statistical testing. ANOVA Tukey test was used to test difference between the dipping groups in continuous variables, and Pearson’s Chi-square test was used for the categorical variables. Delta variables were formed by subtraction.

The Kaplan–Meier method was used to assess hazard of CV events by dipping status categories and tested by log-rank test. We used Cox regression analysis to estimate the association of the dipping status categories with CV events in multivariate models adjusted by variables with significance in univariate analyses. The covariates (change of dipping status, age, sex, total cholesterol, hypertension, use of antihypertensive medication, office blood pressure, and either 24-h mean SBP or nighttime mean SBP) were selected according to the univariate analyses (shown as Supplementary Tables 1 and 2). Categorical covariates included dipping status (dipping–dipping was the reference), sex (women as a reference), hypertension (no hypertension as a reference) and use of antihypertensive medication (medication as a reference). Hypertension was defined by office BP over 140/90 or use of antihypertensive medication. The other covariates were used as continuous variables. Total cholesterol was selected to the model out of the lipids. If it was replaced by any of the lipids (LDL-cholesterol, HDL-cholesterol, or triglycerides), the significance of the mode, or the dipping status, did not change.

## Results

**Fig. 1:**
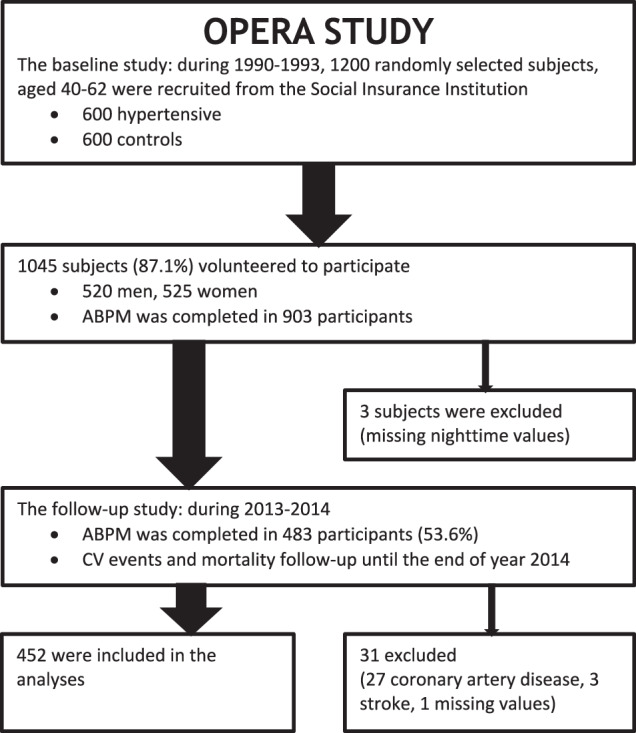
Flow chart of the study population of the OPERA study. Inclusion and exclusion of study subjects, resulting in 452 eligible participants. ABPM Ambulatory blood pressure monitoring; CV cardiovascular.

Our study population consisted of the OPERA study cohort whose participants underwent ABPM in the baseline study and again 21.7 years later (SD ± 0.7, range 20.4–23.8 years) during the follow-up study (Fig. 1). Twenty-seven persons were excluded from the analyses based on an earlier diagnosis of coronary artery disease, three persons because of a history of previous stroke, and one because of missing nighttime ABPM values.

In the analyses, there were altogether 452 eligible individuals, of which 228 (50.4%) were women and 224 (49.6%) men (Table 1). During the recruitment, the mean age of the participants was 49.7 ± 5.4 years, and the age range was from 40.2 to 62.0 years. Non-fatal CV events (*n* = 65, in 14.4% of the subjects) were assessed during the follow-up until the first event. As expected, those who experienced a CV event were predominantly men (*p* < 0.001), older (*p* = 0.012), had higher 24-h mean systolic (*p* = 0.013), daytime systolic (*p* = 0.033), nighttime systolic (*p* < 0.001) and nighttime diastolic BP (*p* = 0.001) and more unfavorable lipid profile than those without a CV event. Almost half of the subjects were hypertensive (47.6%), and 45.4% were treated with at least one antihypertensive medication. The prevalence of diabetes was 6.9%. The average body mass index was 27.2 ± 4.3 kg/m^2^. Of all the subjects, 22.6% were non-dippers. The prevalence of non-dippers was significantly higher in those with a CV event than in those without (40.0% vs. 19.6%, *p* < 0.001) (Table 1).

**Table 1:** The baseline characteristics of the study population and CV events.

	All *n* = 452	Without CV event *n* = 387	CV event *n* = 65	*p* value
Age, year	49.7 ± 5.4	49.4 ± 5.4	51.2 ± 5.4	0.012
Females/Males, *n* (%)	228 (50.4)/224 (49.6)	211 (54.5)/176 (45.5)	17 (26.2)/48 (73.8)	<0.001
BMI, kg/m^2^	27.2 ± 4.3	27.1 ± 4.4	27.4 ± 3.6	0.583
Alcohol consumption, g/week	24 (3–82)	24 (3–72)	45 (6–125)	0.67
Smoking, *n* (%)	109 (24.1)	93 (24.0)	16 (24.6)	0.919
HTA, *n* (%)	215 (47.6)	178 (46.0)	37 (56.9)	0.103
DM, *n* (%)	31 (6.9)	26 (6.7)	5 (7.7)	0.774
Office SBP, mmHg	145 ± 21	144 ± 21	149 ± 19	0.058
Office DBP, mmHg	88 ± 12	88 ± 12	90 ± 11	0.087
ABPM				
24-h SBP, mmHg	127 ± 12	127 ± 12	131 ± 14	0.013
24-h DBP, mmHg	81 ± 8	81 ± 8	82 ± 8	0.122
Daytime SBP, mmHg	133 ± 13	132 ± 12	136 ± 15	0.033
Daytime DBP, mmHg	85 ± 8	85 ± 9	86 ± 9	0.357
Nighttime SBP, mmHg	115 ± 14	114 ± 13	120 ± 14	<0.001
Nighttime DBP, mmHg	70 ± 9	69 ± 9	73 ± 9	0.001
Non-dipper, *n* (%)	102 (22.6)	76 (19.6)	26 (40.0)	<0.001
hsCRP, mg/l	1.2 (0.6–2.5)	1.2 (0.6–2.4)	1.2 (0.7–2.6)	0.68
eGFR, ml/min/1.73 m^2^	85 ± 14	85 ± 14	86 ± 13	0.734
Fasting glucose, mmol/l	4.6 ± 1.3	4.5 ± 1.2	4.7 ± 1.5	0.331
Fasting insulin, mU/l	10.0 (6.8–14.6)	9.9 (6.8–14.5)	10.4 (7.4–14.7)	0.381
Total cholesterol, mmol/l	5.6 ± 1.0	5.6 ± 0.9	5.9 ± 1.3	0.006
Triglycerides, mmol/l	1.2 (0.9–1.7)	1.2 (0.9–1.7)	1.4 (1.1–2.3)	<0.001
HDL-cholesterol, mmol/l	1.4 ± 0.4	1.4 ± 0.4	1.2 ± 0.3	0.001
LDL-cholesterol, mmol/l	1.5 ± 0.9	3.4 ± 0.9	3.7 ± 1.1	0.016
Antihypertensive medication, *n* (%)	205 (45.4)	169 (43.7)	36 (55.4)	0.079
Beta blocker, *n* (%)	109 (24.1)	93 (24.0)	16 (24.6)	0.919
Diuretic, *n* (%)	56 (12.4)	47 (12.1)	9 (13.8)	0.700
CCB, *n* (%)	37 (8.2)	28 (7.2)	9 (13.8)	0.072
ACEi, *n* (%)	87 (19.2)	70 (18.1)	17 (26.2)	0.127
Other, *n* (%)	13 (2.9)	11 (2.8)	2 (3.1)	0.583
Lipid medication, *n* (%)	11 (2.4)	9 (2.3)	2 (3.1)	0.716

Data are presented as prevalence or as mean ± SD for continuous variables, or as median (25th–75th) for skewed variables. Differences between groups were tested with Student’s *t* test, and Kruskal–Wallis test was used for skewed variables.

*BMI* body mass index, *HTA* hypertension, *DM* diabetes, *SBP* systolic blood pressure, *DBP* diastolic blood pressure, *ABPM* ambulatory blood pressure measurement, *hsCRP* highly sensitive C reactive protein, *eGFR* estimated glomerular filtration rate, *HDL* high density lipoprotein, *LDL* low density lipoprotein, *CCB* calcium channel blocker, *ACEi* angiotensin-converting enzyme inhibitor.

**Fig. 2:**
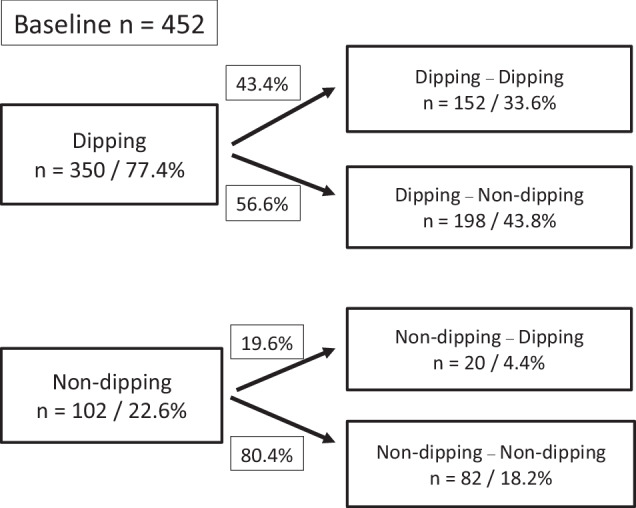
Flow chart of the cohort (*n* = 452) from the baseline study to the follow-up by the dipping status.

The cohort was divided into four groups according to the dipping status change from the baseline to the follow-up study (Fig. 2). Dipping–dipping pattern was present in 33.6% of the whole cohort, and non-dipping–non-dipping pattern in 18.2%. Non-dipping phenotype was the most consistent: it prevailed in the follow-up in 80.4% of the baseline non-dippers, whereas 43.4% of the baseline dippers remained dippers. A large proportion of the original dippers changed their phenotype to non-dippers (56.6%).

Altogether, 65 non-fatal CV events occurred, and the incidence was the highest (28.0%) in the non-dipping–non-dipping group and the lowest (6.6%) in the dipping–dipping group, *p* < 0.001 (Table 2). Age, office BPs, 24-h mean BPs and nighttime BPs differed between the groups and were significantly higher in the non-dipping–non-dipping group compared with the dipping–dipping group. The non-dipping–non-dipping group also had lower estimated glomerular filtration rate (eGFR), total cholesterol, LDL cholesterol and triglycerides levels compared with the dipping–dipping group. Additionally, there was a trend of rising prevalence of hypertension (*p* < 0.001) and antihypertensive medication use (*p* < 0.001) in the non-dipping–non-dipping group (linear by linear association *p* < 0.001).

**Table 2:** The baseline characteristics of the study population by the dipping categories.

	Dipping–Dipping *n* = 152	Dipping–Non-dipping *n* = 198	Non-dipping–Dipping *n* = 20	Non-dipping–Non-dipping *n* = 82	*p* value
CV event, *n* (%)	10 (6.6)	29 (14.6)	3 (15.0)	23 (28.0)	<0.001
Age, year	48.7 ± 5.1	50.1 ± 5.6	49.5 ± 65.5	50.6 ± 5.1	0.031 c*
Females/Males, *n* (%)	88 (57.9)/64 (42.1)	90 (45.5)/108 (54.5)	7 (35.0)/13 (65.0)	43 (52.4)/39 (47.6)	0.061
BMI, kg/m^2^	26.9 ± 4.0	26.8 ± 4.2	26.8 ± 3.3	28.7 ± 5.2	0.06 c*, e*
Alcohol consumption, g/week	24 (23–72)	24 (3–82)	23 (2–80)	31 (5–144)	0.67
Smoking, *n* (%)	39 (25.7)	52 (26.3)	2 (10.0)	16 (19.5)	0.281
HTA, *n* (%)	57 (37.5)	89 (44.9)	12 (60.0)	57 (69.5)	<0.001
DM, *n* (%)	9 (5.9)	12 (6.1)	2 (10.0)	8 (9.8)	0.616
Office SBP, mmHg	141 ± 19	145 ± 21	148 ± 16	150 ± 21	0.007 c**
Office DBP, mmHg	86 ± 12	88 ± 12	94 ± 8	91 ± 13	0.001 b*, c**
ABPM					
24-h SBP, mmHg	125 ± 12	128 ± 12	133 ± 13	130 ± 13	0.002 b*, c*
24-h DBP, mmHg	79 ± 8	81 ± 8	87 ± 8	82 ± 8	<0.001 b**, d*
Daytime SBP, mmHg	131 ± 12	134 ± 13	135 ± 14	132 ± 14	0.175
Daytime DBP, mmHg	84 ± 8	85 ± 9	88 ± 9	84 ± 9	0.078
Nighttime SBP, mmHg	109 ± 11	113 ± 11	128 ± 13	125 ± 13	<0.001 a*, b–e***
Nighttime DBP, mmHg	66 ± 8	69 ± 8	80 ± 8	76 ± 9	<0.001 a*, b–e***
hsCRP, mg/l	0.9 (0.6–2.2)	1.3 (0.7–2.4)	1.1 (0.3–2.3)	1.5 (0.8–3.5)	0.051 c*
eGFR, ml/min/1.73 m^2^	87 ± 13	87 ± 14	82 ± 12	82 ± 15	0.019 c*, e*
Fasting glucose, mmol/l	4.5 ± 1.1	4.5 ± 1.3	4.3 ± 0.6	4.7 ± 1.7	0.452
Fasting insulin, mU/l	9.3 (6.4–13.4)	9.9 (6.8–13.8)	10.8 (6.4–13.6)	10.8 (7.3–17.1)	0.155
Total cholesterol, mmol/l	5.5 ± 0.9	5.6 ± 1.0	5.3 ± 0.8	5.9 ± 1.0	0.007 c*, f*
Triglycerides, mmol/l	1.2 (0.9–1.6)	1.2 (0.9–1.7)	1.0 (0.8–1.4)	1.4 (1.1–2.2)	0.004 c**
HDL-cholesterol, mmol/l	1.4 ± 0.4	1.4 ± 0.4	1.3 ± 0.5	1.3 ± 0.3	0.210
LDL-cholesterol, mmol/l	3.4 ± 0.9	3.5 ± 0.9	3.2 ± 0.9	3.7 ± 0.9	0.053
Any antihypertensive agent, *n* (%)	53 (34.9)	87 (43.9)	11 (55.0)	54 (65.9)	<0.001
Beta blocker, *n* (%)	30 (19.7)	45 (22.7)	6 (30.0)	28 (34.1)	0.083
Diuretic, *n* (%)	13 (8.6)	29 (14.6)	0	14 (17.1)	0.058
CCB, *n* (%)	9 (5.9)	11 (5.6)	4 (20.0)	13 (15.9)	0.005
ACEi, *n* (%)	24 (15.8)	32 (16.2)	5 (25.0)	26 (31.7)	0.012
Other, *n* (%)	4 (2.6)	5 (2.5)	1 (5.0)	3 (3.7)	0.891
Lipid medication, *n* (%)	1 (0.7)	7 (3.5)	1 (5.0)	2 (2.4)	0.31

Data are presented as prevalence or as mean ± SD for continuous variables, or as median (25th–75th) for skewed variables. Differences between groups were tested with ANOVA. Kruskal–Wallis test was used for skewed variables. Post hoc analysis was used to analyze the differences between the groups: 1–2 = a, 1–3 = b, 1–4 = c, 2–3 = d, 2–4 = e, 3–4 = f, level of significance: *p < 0.05; **p < 0.01; ***p < 0.001.

*CV* cardiovascular, *BMI* body mass index, *HTA* hypertension, *DM* diabetes, *SBP* systolic blood pressure, *DBP* diastolic blood pressure, *ABPM* ambulatory blood pressure monitoring, *hsCRP* high sensitive C reactive protein, *eGFR* estimated glomerular filtration rate, *HDL* high density lipoprotein, *LDL* low density lipoprotein, *CCB* calcium channel blocker, *ACEi* angiotensin-converting enzyme inhibitor.

**Fig. 3:**
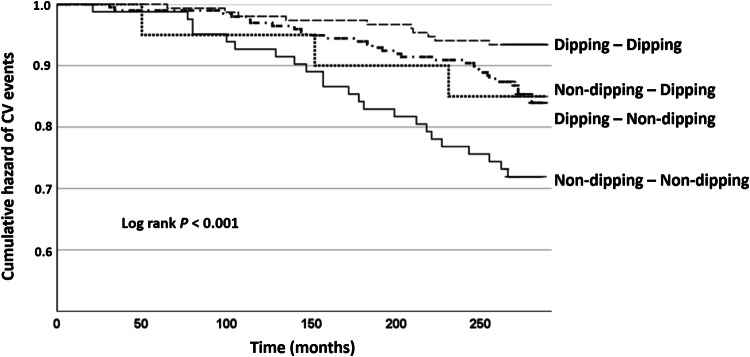
Kaplan–Meier survival curves of CV events by the dipping status groups.

Kaplan–Meier survival curves for CV events by different dipping patterns are presented in Fig. 3.

We analyzed the changes in BP, metabolic factors, and prevalence of hypertension and medication use during the follow-up time (Table 3) by the dipping categories. In the non-dipping–non-dipping group, the incidence of new-onset diabetes was significantly higher than in the dipping–dipping group, and it was reported earlier [11].

**Table 3:** Changes in variables during the follow-up time shown in respective units or incidence.

	Dipping–Dipping *n* = 152	Dipping–Non-dipping *n* = 198	Non-dipping–Dipping *n* = 20	Non-dipping–Non-dipping *n* = 82	*p* value
CV event, *n* (%)	10 (6.6)	29 (14.6)	3 (15.0)	23 (28.0)	<0.001
BMI, kg/m^2^	+1.3	+2.1	+1.9	+1.1	0.071
Alcohol consumption, g/week	−11.4	−13.8	+21.5	−24.8	0.056
Smoking, pack year	+3.3	+3.2	+0.4	+2.0	0.448
New HTA	+41 (27.0)	+56 (28.3)	+7 (35.0)	+13 (15.9)	0.119
New DM	+29 (19.1)	+57 (28.8)	+3 (15.0)	+31 (37.8)	0.009
Office SBP, mmHg	+0.1	−7.0	−6.3	−17.8	<0.001
Office DBP, mmHg	−11.7	−16.0	−17.5	−20.7	<0.001
ABPM					
24-h SBP, mmHg	+5.5	+5.4	−2.1	+4.3	0.275
24-h DBP, mmHg	−7.3	−8.4	−13.2	−8.3	0.09
Daytime SBP, mmHg	+3.0	+0.4	−0.5	+2.7	0.462
Daytime DBP, mmHg	−9.8	−12.0	−12.5	−9.7	0.11
Nighttime SBP, mmHg	+3.8	+17.8	−12.8	+7.9	<0.001
Nighttime DBP, mmHg	−6.2	−0.4	−19.6	−6.0	<0.001
eGFR, ml/min/1.73 m^2^	−2.4	−3.7	−2.3	−2.5	0.797
Fasting glucose, mmol/l	+1.63	+1.68	+1.57	+1.49	0.698
Total cholesterol, mmol/l	−0.58	−0.88	−0.63	−1.24	0.004
Triglycerides, mmol/l	−0.06	−0.09	0.00	−0.25	0.254
HDL, mmol/l	+0.16	+0.08	+0.03	+0.09	0.094
LDL, mmol/l	−0.36	−0.62	−0.36	−0.83	0.035
New onset medication, *n* (%)					
Antihypertensive agent	+47 (30.9)	+70 (35.4)	+7 (35.0)	+18 (22.0)	0.174
Beta blocker	+36 (23.7)	+57 (28.8)	+4 (20.0)	+26 (31.7)	0.48
Diuretic	+42 (27.6)	+50 (25.3)	+5 (25.0)	+32 (39.0)	0.13
CCB	+30 (19.7)	+68 (34.3)	+4 (20.0))	+27 (32.9)	0.015
ACEi	+21 (13.8)	+31 (15.7)	+1 (5.0)	+11 (13.4)	0.615
ARB	+36 (23.7)	+55 (27.8)	+9 (45.0)	+28 (34.1)	0.124

Data are presented as prevalence or as mean ± SD for continuous variables, or as median (25th–75th) for skewed variables. Differences between groups were tested with ANOVA *t*-test, and Kruskal–Wallis test was used for skewed variables.

*CV* cardiovascular, *BMI* body mass index, *HTA* hypertension, *DM* diabetes, *SBP* systolic blood pressure, *DBP* diastolic blood pressure, *ABPM* ambulatory blood pressure monitoring, *eGFR* estimated glomerular filtration rate, *HDL* high density lipoprotein, *LDL* low density lipoprotein, *CCB* calcium channel blocker, *ACEi* angiotensin-converting enzyme inhibitor, *ARB* angiotensin receptor blocker.

A general positive development over time in BP measurements can be observed, with the DBP decreasing more, as expected, with age. There were statistically significant differences in office BP and nighttime BP between the dipping groups. In the non-dipping–non-dipping group, the reduction of office BP was the greatest and in the dipping–dipping group the smallest. In nighttime mean BP, the groups also differed from each another, but in post hoc analyses, there was no statistically significant difference in the change in the nighttime BP between the dipping–dipping and the non-dipping–non-dipping group.

As expected, the prevalence of any BP medication use increased over time in all the dipping groups, especially the proportions of the RAA system agents. The non-dipping–non-dipping group had more often BP medication than the dipping–dipping group (86.6% vs. 63.2%, *p* < 0.001 between the groups, data shown as Supplementary Table 3).

In the follow-up, a lipid lowering agent was used by 45.4% of the study population, compared to only 2.4% at the baseline. Non-dippers tended to use statins more often than the other groups in the follow-up, but the difference did not reach statistical significance.

In the multivariate Cox model, we included variables, that were statistically significant in univariate analyses (data shown only as Supplementary Tables 1 and 2): dipping change pattern (dipping–dipping group as a reference), age, sex, hypertension, office SBP, 24-h mean SBP or nighttime mean SBP, total cholesterol and use of antihypertensive agent (Table 4). The baseline fasting blood glucose levels, prevalence of diabetes or smoking did not differ between the dipping groups and were therefore left out of the Cox model. The non-dipping–non-dipping pattern was independently associated with CV events when adjusted with the above covariates and the 24-h mean SBP (HR 4.01; 95% CI 1.86–8.67, *p* < 0.001) (Table 4a). The association between the non-dipping–non-dipping pattern and CV events also remained statistically significant when the adjustment in this model was made with the nighttime mean SBP instead of the 24-h mean SBP (HR 3.19; 95% CI 1.41–7.20, *p* = 0.005) (Table 4b).

**Table 4:** Multivariate Cox regression models: association of the dipping pattern and CV events.

	HR	95% Cl	*p* value
(a) Multivariate Cox regression: association of the dipping pattern and CV events, adjusted with 24-h mean SBP			
Dipping–Dipping (Ref.)			0.002
Dipping–Non-dipping	1.759	0.849–3.646	0.129
Non-dipping–Dipping	1.697	0.455–6.324	0.431
Non-dipping–Non-dipping	4.012	1.856–8.672	<0.001
Age	1.053	1.006–1.103	0.028
Sex (F = Ref.)	2.933	1.647–5.223	<0.001
Total cholesterol	1.261	0.974–1.631	0.078
Hypertension	0.812	0.277–2.377	0.704
Antihypertensive medication	0.685	0.245–1.92	0.472
Office SBP	0.993	0.979–1.007	0.347
24-h mean SBP	1.021	0.997–1.047	0.088
(b) Multivariate Cox regression: association of the dipping pattern and CV events, adjusted with nighttime mean SBP			
Dipping–Dipping (Ref.)			0.030
Dipping–Non-dipping	1.720	0.828–3.571	0.146
Non-dipping–Dipping	1.366	0.348–5.365	0.655
Non-dipping–Non-dipping	3.190	1.414–7.197	0.005
Age	1.053	1.005–1.102	0.029
Sex (F = Ref.)	2.898	1.626–5.165	<0.001
Total cholesterol	1.263	0.975–1.636	0.076
Hypertension	0.848	0.294–2.442	0.760
Antihypertensive medication	0.723	0.262–1.996	0.531
Office SBP	0.993	0.979–1.008	0.360
Nighttime mean SBP	1.021	0.996–1.047	0.100

*Ref.* reference, *F* female, *SBP* systolic blood pressure.

Finally, the delta values of nighttime SBP or 24-h mean SBP, as well as delta office SBP, total cholesterol, new onset diabetes, new onset hypertension, and the change in BP medication were added to multivariate models. The statistical significance of the association of the dipping pattern with CV events remained (for nighttime SBP delta: HR 3.40; 95% CI 1.47–7.88, *p* = 0.004 and for 24-h mean SBP: HR 3.13; 95% CI 1.38–7.11, *p* = 0.006).

## Discussion

This population-based study consisted of 452 originally middle-aged Finnish hypertensive and normotensive females and males who underwent ABPM at the baseline and at the end of the follow-up time, 21.7 years later. The prevalence of non-dipping varies among studies, from 20% in white young adult women [19] to 86% in CKD patients [20]. In this study, at the baseline, 22.6% were non-dippers. Non-dipping–non-dipping pattern of nighttime BP, which was detected in 80.4% of the original non-dippers, was associated with non-fatal CV events during the follow-up even when adjusting with demographics, the classic CV risk factors, and either 24-h mean SBP or nighttime SBP.

In the present study, the association of the non-dipping status and non-fatal CV events was not explained by the unfavorable development of the conventional risk factor levels or the differences in them between the groups. In our previous study, we showed that the non-dipping–non-dipping group developed new-onset diabetes more often than the dipping–dipping group [11]. However, in the present study, all the multivariate analyses were controlled for diabetes as well as other risk factors. The trend toward lower risk factor levels of several variables during the follow-up period was more enhanced in the non-dipping–non-dipping group: BP declined more in non-dippers than dippers, but only the difference in the office BP reached statistical significance. Also, lipid levels improved in general, and the change was more prominent in non-dippers than in dippers. Furthermore, the use of antihypertensive and lipid lowering agents was more prevalent in the non-dipping–non-dipping group than in the dipping–dipping group. Nevertheless, the CV risk was greater in those with non-dipping–non-dipping. In a cohort of hypertensive middle-aged patients, with ABPM seven years apart, the unmedicated baseline ABPM predicted future CV events better than the latter recording in a 10-year follow-up [21] .

Our study has strengths, but also limitations. One strength is that the nighttime BP was measured three times per hour. If BP is recorded only once or twice an hour, the value of hourly mean BP is rapidly diminished [22]. Other strengths are the comprehensive data of the population and hospital records, which are available and collectable by a personal identity code provided to every citizen, as well as high participation percentages in both our surveys.

There are also limitations: one is that the reproducibility of BP phenotypes identified by both office BP and ABP has been questioned. According to a recent review, in hypertensive patients on antihypertensive medication, only a limited number of individuals maintained the same dipping pattern over a few years of follow-up, unrelated to the type of antihypertensive medication used [23]. On the contrary, there is also data of higher agreement between ABPM recordings 2.5 years apart and a coherence of up to 76% [24]. Nighttime BP has been associated with poor CV outcome both in population studies and in hypertensive persons [25], and overall reproducibility is considered the best in the majority of subjects with the non-dipping pattern of nocturnal BP [26].

The day-to-day variation of dipping status has been suspected of being to some extent a result of sleep quality [27]. In this study we did not collect data regarding the quality of sleep or the prevalence of sleep apnea. Instead of diary records, fixed-clock intervals were used in defining daytime and nighttime, and the transitional times in the morning and in the evening were not excluded from the analyses. In a 17-year-long study, however, the researchers stated that while diary records are preferable, standard fixed-time intervals are also suitable in population-based studies [28]. Furthermore, we do not have data on timing of the antihypertensive medication which was common among our study population.

To conclude, the non-dipping–non-dipping pattern of BP was independently associated with non-fatal CV events in a randomly selected normotensive and hypertensive middle-aged population in a two-decade follow-up study.

## Summary

### What is known about the topic

- Non-dipping blood pressure pattern is associated with cardiovascular events, end-organ damage, and metabolic abnormalities.
- Nocturnal BP levels may be better to predict CV events and mortality than office BP.

### What this study adds

- Non-dipping blood pressure pattern was the most consistent during the 21-year follow-up. Eighty-two (80.2%) out of the 102 baseline non-dippers remained non-dippers, whereas 43.4% (*n* = 152) of the baseline dippers remained dippers.
- Non-dipping–non-dipping pattern in the long term was associated with increased risk of non-fatal CV events compared to dipping–dipping pattern.

### Supplementary information

Supplemental Table 1

Supplemental Table 2

Supplemental Table 3

## Data Availability

Data cannot be shared publicly because of privacy policy. Data are available from the Oulu University Institutional Data Access for researchers who meet the criteria for access to confidential data. Contact information for the Oulu University Institutional Data Access committee: olavi.ukkola@oulu.fi.
